# A Single Gradient Stability-Indicating Reversed-Phase LC Method for the Estimation of Impurities in Omeprazole and Domperidone Capsules

**DOI:** 10.3797/scipharm.1209-12

**Published:** 2012-11-19

**Authors:** Raja Kumar Seshadri, Thummala Veera Raghavaraju, Ivon Elisha Chakravarthy

**Affiliations:** 1 Analytical Research and Development, Integrated Product Development, Dr. Reddy’s Laboratories Ltd., Bachupally, Hyderabad-500 072, India.; 2 Department of Chemistry, Rayalaseema University, Karnool-518 002, A.P., India.

**Keywords:** Omeprazole, Domperidone, RP-LC, Method validation, Forced Degradation

## Abstract

A gradient reversed-phase liquid chromatographic (RP-LC) method was developed for the quantitative estimation of impurities in the pharmaceutical dosage form of Omeprazole and Domperidone capsules. The developed method is a stability-indicating test method for the estimation of impurities generated during the formulation and storage of Omeprazole and Domperidone capsules. The chromatographic separation was achieved on a column packed with octadecyl silane, having a column length of 250 mm and diameter of 4.6 mm with a particle size of 5 μm, and by following a gradient program using a combination of a monobasic potassium phosphate buffer (0.05M) and acetonitrile. Since the spectral properties were similar, both compounds’ individual impurities were estimated at 285 nm. Forced degradation studies were performed on Omeprazole pellets (enteric coated) and Domperidone pellets (SR coated) encapsulated in size ‘1’ hard gelatin capsules. Omeprazole and Domperidone were degraded using acid hydrolysis (0.1 N hydrochloric acid), base (0.1 N sodium hydroxide), oxidation (50% hydrogen peroxide), heat (105 °C), and UV light (254 nm). The established method was validated and found to be linear, accurate, precise, specific, robust, and rugged.

## Introduction

**Fig. 1:**
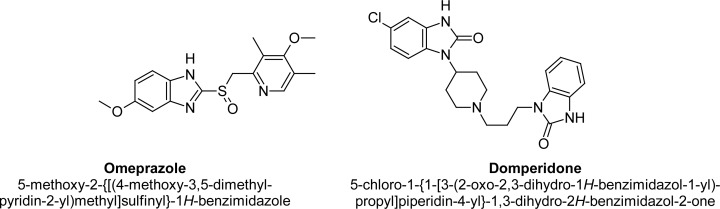
Chemical structures of OM and DP

The combination of Omeprazole (OZ) and Domperidone (DP) is used for duodenal ulcers, gastric ulcers, reflux, or ulcerative esophagitis, etc. OZ is a class of medications called proton pump inhibitors [1, 2]. It suppresses gastric acid secretion through the specific inhibition of the H^+^/K^+^-ATPase enzyme system at the secretory surface of the gastric parietal cell [1]. By acting specifically on the proton pump, OZ blocks the final step in acid production, thus reducing the gastric acidity. DP is a dopamine receptor antagonist, which works as an upper gastrointestinal prokinetic and increases the tone of the lower esophageal sphincter and enhances gastric emptying [3, 4]. It does not produce dopamine antagonist effects in the CNS, probably because it fails to cross the blood brain barrier. It facilitates gastrointestinal smooth muscle activity by inhibiting dopamine at the D1 receptors. The chemical structures of OZ and DP are shown in Fig. 1.

**Fig. 2:**
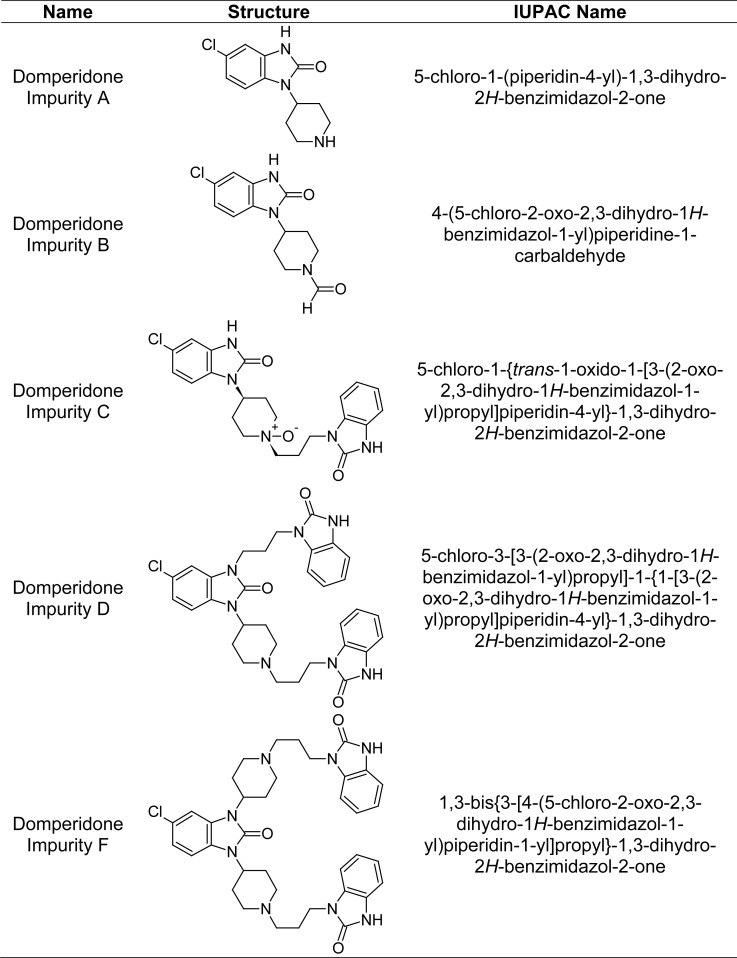

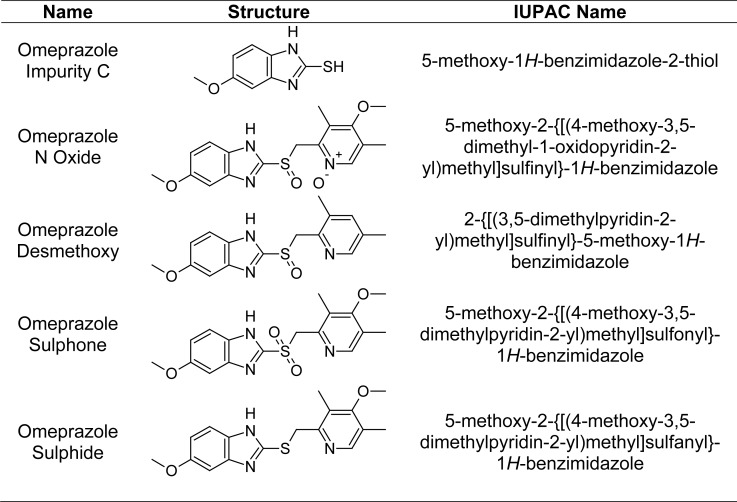
Chemical structures of impurities

So far, various reported RP-LC (HPLC) methods [5–7] include the estimation of impurities in OZ and OZ capsules, but none of the methods described a single method for the estimation of impurities in the combination product of OZ and DP capsules. The present paper describes the quantitative estimation of impurities in the combination product. This paper also deals with the forced degradation study under various stress conditions such as acid hydrolysis, base hydrolysis, oxidation, heat, and UV and also method validation [8] for the accurate quantification of known impurities in pharmaceutical formulations. The chemical structures of impurities are shown in Fig. 2.

## Experimental

### Chemicals

Active pharmaceutical ingredient samples of OZ and DP as well as impurities were received from bulk manufacture at Dr. Reddy’s Laboratories Limited, Hyderabad, India and BP and Sidmak. Capsules were received from Sidmak Laboratories. HPLC grade acetonitrile was purchased from Merck, Germany. Analytical reagent monobasic potassium phosphate and sodium hydroxide were purchased from Merck, Germany. High purity water was prepared by using the Millipore Milli-Q Plus purification system.

### Equipment

The Waters RP-LC (HPLC) system with a diode array detector was used for the method development and forced degradation studies. The output signal was monitored and processed using Millenium software.

The LC (HPLC) system used for method validation was the Agilent HPLC. The output signal was monitored and processed using HP ChemStation software (Agilent) on a Pentium computer.

### Chromatographic conditions

The chromatographic column used was the Inertsil ODS 3V 250 × 4.6mm with 5 μm particles. The buffer used was 0.05M monobasic potassium phosphate pH-adjusted to 7.2 using a 0.1N sodium hydroxide solution. The buffer and acetonitrile in the ratio of 75:25 was used as mobile phase A, and the buffer and acetonitrile in the ratio of 45:55 were used as mobile phase B. The flow rate of the mobile phase was 1.2 mLmin^−1^. The column was maintained at 25 °C and the wavelength of 285 nm was used for the detection of impurities in both compounds. The injection volume was 20 μL.

### Diluent

0.1N sodium hydroxide was used as diluent.

### Gradient Program

**Table:** 

**Time**	**Flow**	**% A**	**% B**
0	1.2	100	0
20	1.2	100	0
30	1.2	25	75
40	1.2	25	75
50	1.2	100	0
55	1.2	100	0

### Preparation of standard solution

**Fig. 3:**
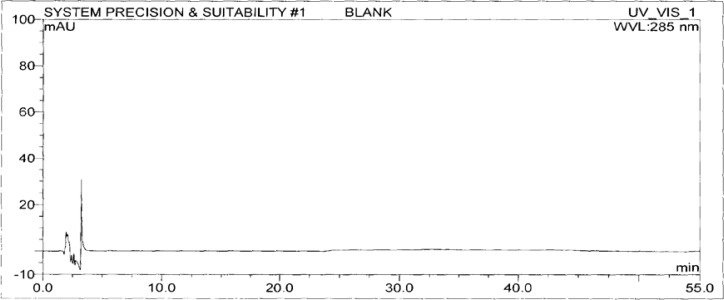
The typical chromatogram of Diluent

**Fig. 4:**
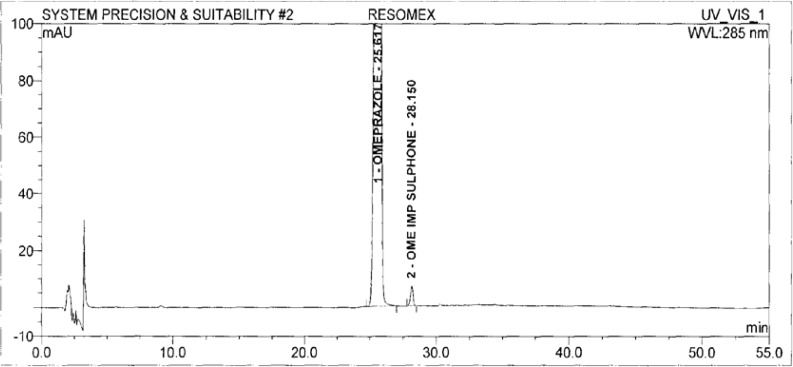
The typical chromatogram of Standard

A standard stock solution of OZ and DP (0.5mgmL^−1^ and 0.75mgmL^−1^ respectively) was prepared by dissolving an appropriate amount of substance in diluent. The stock solution was further diluted with diluent to obtain a standard solution of 3 μgmL^−1^ and 4.5 μgmL^−1^ respectively for the determination of impurities. The typical chromatograms of the standard and diluent are shown in Fig. 3 and Fig. 4.

### Preparation of Test solution

**Fig. 5:**
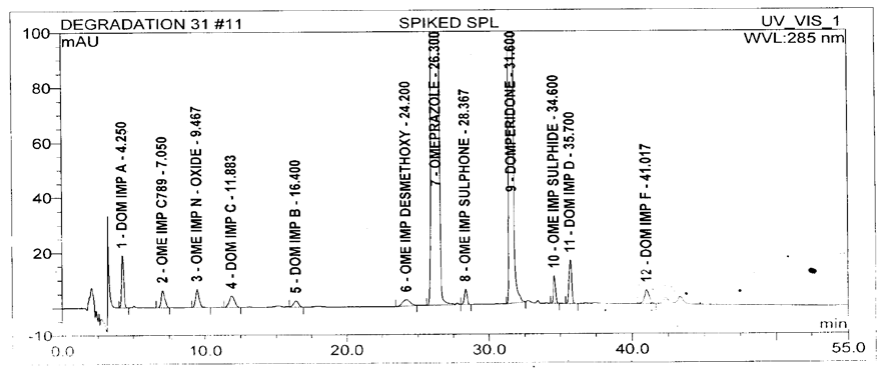
The typical chromatogram of Test preparation with spiked impurities

**Fig. 6:**
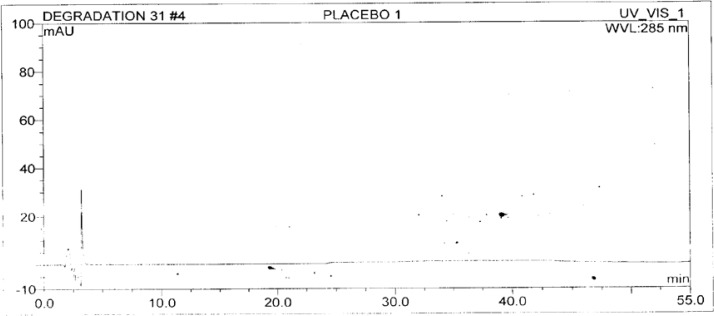
The typical chromatogram of Placebo

The test solution was prepared by taking the pellet powder equivalent to 1.0mgmL^−1^ and 1.5mgmL^−1^ of OZ and DP respectively. The typical chromatograms of the placebo and OZ and DP capsule tests are shown in Fig. 5 and Fig. 6.

### Preparation of System suitability solution

The system suitability solution was prepared with OZ sulphone (3 ppm) with 1.0 mgmL^−1^ solution of OZ. The resolution between the OZ and OZ sulphone impurity was measured.

### Response factor

The measurement of the response factor for each impurity’s determination is important when the calculations are being made on a relative percent basis. Hence, authentic samples of the related substances and R were dissolved in the diluent and injected, and responses were calculated. RRF values are mentioned in Table 1.

## Method validation

### Precision

The precision of the test method was evaluated by analyzing six samples of OZ and DP capsules by spiking the test preparations with the OZ and DP impurities blend solution at 0.3% concentration of each impurity with respect to the test concentration. The relative standard deviation was calculated for the response of each impurity.

### Intermediate Precision

The intermediate precision study was conducted by a different analyst on a different day, column, and on a different LC (HPLC) system. Six samples of OZ and DP capsules were prepared by spiking the test preparations with the OZ and DP impurities blend solution at 0.3% concentration of each impurity with respect to the test concentration. The RSD was calculated for the response of each impurity.

### Limit of detection (LOD) and limit of quantification (LOQ)

The LOD and LOQ of OZ and DP impurities were estimated based on the signal-to-noise ratio to get a signal-to-noise ratio of 3:1 and 10:1, respectively, for each impurity by injecting a series of dilute solutions with known concentration. Precision and accuracy studies were also carried out at the LOQ level by injecting six individual preparations of impurities and calculating the % RSD of the area.

### Linearity

Linearity test solutions for the related substance method were prepared from the impurities stock solution at six concentration levels from the LOQ to 150% of the specification (% of LOQ, 0.15%, 0.225%, 0.3%, and 0.45%). Plotting the peak areas of impurities versus their corresponding concentrations drew the calibration curve. We then calculated the slope and Y-intercept and correlation coefficient of the calibration curve for each impurity.

### Accuracy

A recovery study of OZ and DP impurities from spiked samples of the test preparation was conducted. Samples were prepared in triplicate by spiking the test preparations with 50%, 75%, 100%, 125%, and 150% of the target concentration (0.3%) of OZ and DP impurities. The % recovery of each individual impurity was calculated by the external standard method.

### Specificity

Specificity is the ability of the method to measure the analyte response in the presence of its potential impurities. The specificity of the developed LC (HPLC) method for OZ and DP was carried out in the presence of its related potential impurities.

Forced degradation studies were performed on OZ and DP pellet powders to provide an indication of the stability-indicating property and specificity of the proposed method. Samples were degraded intentionally by stressing samples under UV light (254 nm), heat (60 °C), acid (0.1 N HCl), base (0.1 N NaOH), and oxidation (3% H_2_O_2_) to evaluate the ability of the proposed method to separate OZ, DP, and their known impurities from their degradation products as well as the placebo. For the heat and light studies, the time period for stress was 24 hours, whereas acid, base hydrolysis, and oxidation was 30 minutes. The peak purity test was carried out by using a photodiode array detector for OZ and DP peaks, and the peaks were found to be pure.

### Robustness

To determine the robustness of the developed method, experimental conditions were intentionally altered and the resolution between the OZ sulphone and OZ peaks was evaluated. The flow rate of the mobile phase was 1.2mLmin^−1^. To study the effect of flow rate on the resolution, it was changed by 0.2 units from 1.0 mLmin^−1^ to 1.4 mLmin^−1^, while the other mobile phase components were held constant. The effect of the percent organic strength on the resolution was studied by varying the acetonitrile percentage from −10 to +10%, while the other mobile phase components were held constant. To study the effect of a buffer pH on the resolution, 0.2 units changed it from 7.0 to 7.4, while the other mobile phase components were held constant.

### Solution stability of test preparation

The benchtop solution stability of the test preparation and standard preparation of OZ and DP was carried out up to 48 hours. The standard preparation was found to be stable up to 48 hours, whereas the test preparation was stable only up to 24 hours.

## Results and discussion

### Optimization of chromatographic conditions

OZ Desmethoxy, OZ sulphone, OZ sulphide, OZ N-oxide, and OZ c-789 impurity, DP Impurity A, B, C, D, and F in DP were the potential impurities. The main target of the chromatographic method is to get the separation of all potential impurities in a single chromatographic condition. The separation of all impurities was tried using different mobile phases containing acetate buffers like ammonium acetate and phosphate buffers like potassium dihydrogen phosphate, along with various ratios of organic modifiers like acetonitrile and methanol using a different gradient program. The impurities and degradants pertaining to the individual active moiety are estimated at the specific wavelength of 285 nm.

The chromatographic separation was achieved by the following gradient program using the 0.05M monobasic potassium phosphate buffer, pH adjusted to 7.2 with 0.1N sodium hydroxide solution. The buffer and acetonitrile in the ratio of 75:25 were used as mobile phase A, and the buffer and acetonitrile in the ratio of 45:55 were used as mobile phase B. A gradient program was necessary to elute all the impurities and degradation products with good resolution. The typical retention times of OZ and OZ Desmethoxy, OZ sulphone, OZ sulphide, OZ N-oxide, and OZ c-789 are 25.8 min, 23.2 min, 27.92 min, 34.4 min, 9.16 min, and 7.0 min respectively.

The typical retention times of DP and DP Impurity A, B, C, D, and F are 31.5 min, 4.1 min, 15.8 min, 11.16 min, 35.5 min, and 40.5 min respectively.

System suitability was established as OZ and OZ sulphone peaks were eluting closely. The resolution between OZ and OZ sulphone peaks was found to be more than 2.0. The relative retention time and relative response factors were evaluated for impurities. The developed LC method was found to be specific for OZ and DP and their impurities.

### Results of Forced degradation experiments

Degradation was observed in OZ and DP under stress conditions like UV light, heat, and also acid, base peroxide hydrolysis. Peak purity has been verified for all of the impurities and for both main peaks; the peak purity matches for all impurities and main compounds were found to be more than 990. Peak purity shows that impurity peaks as well as main peaks are homogeneous under all the stress conditions. By the above-mentioned fact we can confirm that the method is a stability-indicating method. The summary of the forced degradation studies and peak purity details are given in Table 2. The chromatograms and purity plots of the stressed samples are shown in Fig. 7 to Fig. 11.

### Precision

The % RSD of the response of all impurities during precision and intermediate precision was found to be less than 10%. The results are shown in Table 3, which indicate good precision of the method

### Limit of detection (LOD) and limit of quantification (LOQ)

The limit of detection (LOD) and limit of quantification (LOQ) for all the impurities were established by the signal-to-noise method, and precision and accuracy is verified at the limit of quantification level. The limit of quantification of the OZ Desmethoxy impurity, OZ sulphone, OZ sulphide, OZ N-oxide, and OZ c-789 are 0.015%, 0.015%, 0.015%, 0.015%, and 0.016% respectively.

The limit of quantification of DP Imp A, B, C, D, and F are 0.005%, 0.015%, 0.015%, 0.005%, and 0.017% respectively

The % recoveries of OZ Desmethoxy impurity, OZ sulphone, OZ sulphide, OZ N-oxide, and OZ c-789 at the LOQ level were found to be 105.1%, 95.7%, 100.5%, 99.6%, and 102.3% respectively.

The % recoveries of DP Imp A, B, C, D, and F at the LOQ level were found to be 98.7%, 108.6%, 95.5%, 100.4%, and 98.3% respectively.

The precision of OZ Desmethoxy impurity, OZ sulphone, OZ sulphide, OZ N-oxide, and OZ c-789 and of DP Imp A, B, C, D, and F at the LOQ level was indicated by the RSD of the response, which was below 15%. The concentration of impurities at the LOQ is summarized in Table 3.

### Linearity

**Fig. 12:**
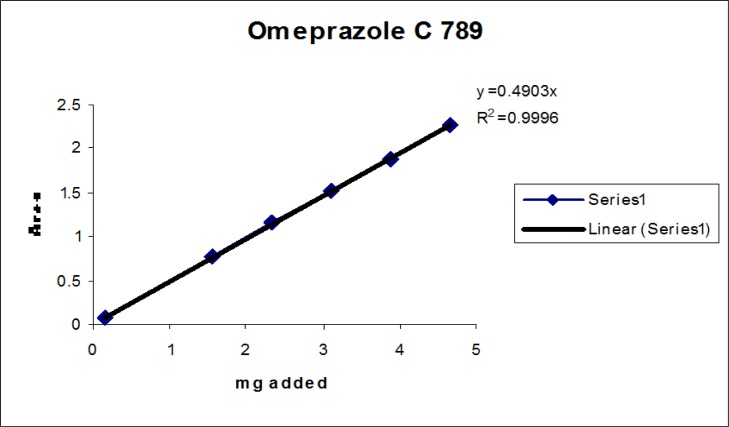
Linearity plot of Omeprazole C 789

**Fig. 21:**
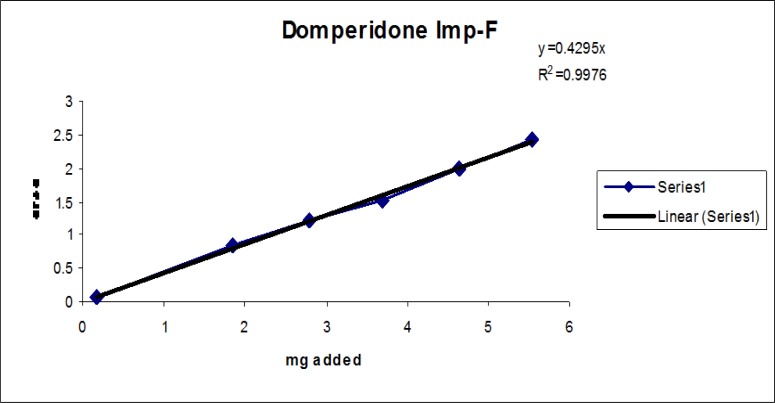
Linearity plot of Domperidone Imp-F

The linear calibration plot for the impurities method was obtained over the calibration ranges tested, i.e. LOQ to 150% of target concentration (0.3%) of individual impurities for all impurities. The correlation coefficient was measured from each impurity’s linear calibration plot and found to be greater than 0.997. The results showed that a good correlation existed between the peak area and concentration of impurities. The results are shown in Fig. 12 to Fig. 21

### Accuracy

The recovery studies were performed from 50 % to 150% of the target concentration (0.3%). The % of mean recovery and % RSD of individual impurities of OZ and DP from the formulation samples were found to be satisfactory.

The mean recovery and RSD of the OZ Desmethoxy impurity was 99.42% and 2.46%. The mean recovery and RSD of OZ sulphone was 103.44 % and 5.12%. The mean recovery and RSD of OZ sulphide was 107.64% and 2.8%. The mean recovery and RSD of OZ N-oxide was 96.15% and 1.81%. The mean recovery and RSD of OZ c-789 was 97.07% and 1.58%.

The mean recovery and RSD of DP Impurity A was 109.98% and 1.87%. The mean recovery and RSD of DP Impurity B was 87.30% and 1.88%. The mean recovery and RSD of DP Impurity C was 99.59% and 2.16%. The mean recovery and RSD of DP Impurity D was 102.73% and 2.4%. The mean recovery and RSD of DP Impurity F was 101.78% and 3.74%. The summary of the % recovery and RSD of individual impurities is mentioned in Table 5.

### Robustness

In all the deliberately varied chromatographic conditions (flow rate, buffer pH, and percentage of organic strength) the resolution between OZ and OZ sulphone peaks was greater than 4.0, which illustrates the robustness of the method.

### Test Solution stability

After 24 hours on the benchtop, no significant change was observed in the % of impurities of DP, whereas a slight variation was observed in the % of impurities of OZ. Hence, the test solutions were freshly prepared and injected into the chromatographic system during method validation.

## Conclusions

The RP-LC method developed for the determination of related substances of OZ and DP is precise, accurate, and selective. The method validation shows satisfactory data for all the method validation parameters tested. The developed method is stability-indicating and can be used for assessing the impurities in OZ and DP capsules and also in their individual dosage forms.

## Figures and Tables

**Fig. 7:**
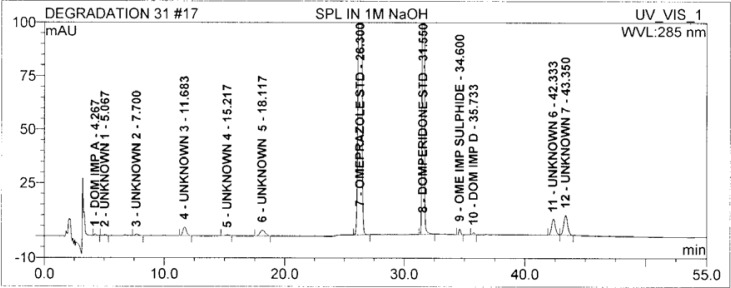
The typical Chromatogram of Sample in 1 M NaOH

**Fig. 7a:**
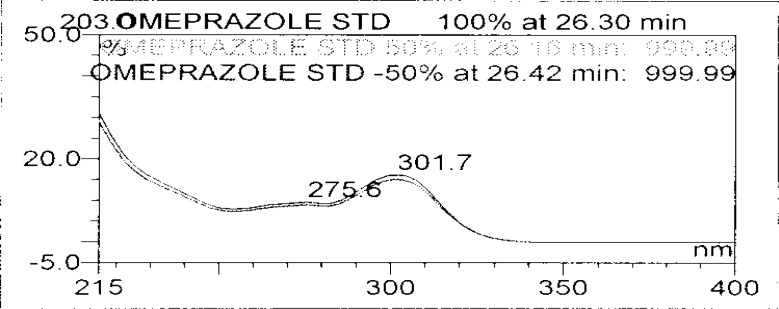
Purity Plot of Omeprazole in 1M NaOH

**Fig. 7b:**
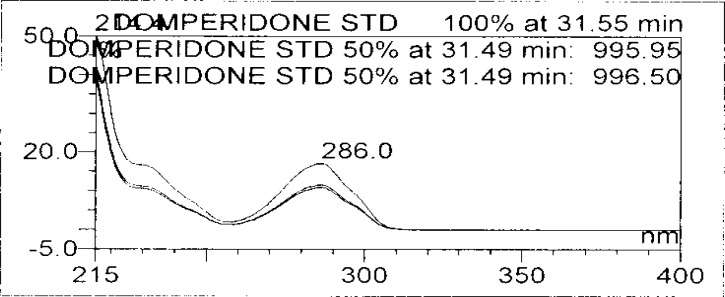
Purity Plot of Domperidone in 1M NaOH

**Fig. 8:**
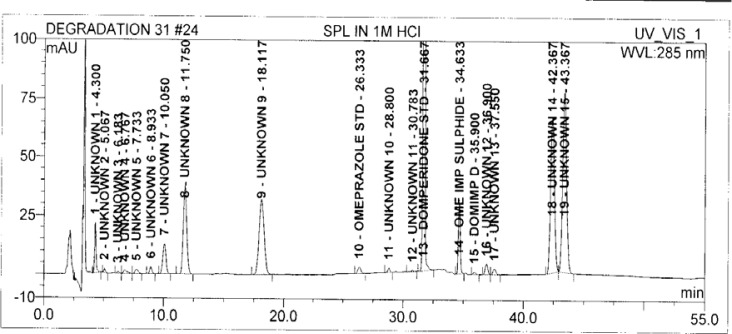
The typical Chromatogram of Sample in 1 M HCl

**Fig. 8a:**
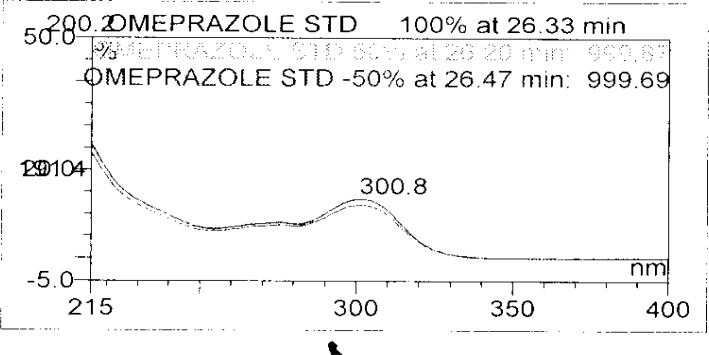
Purity Plot of Omeprazole in 1M HCl

**Fig. 8b:**
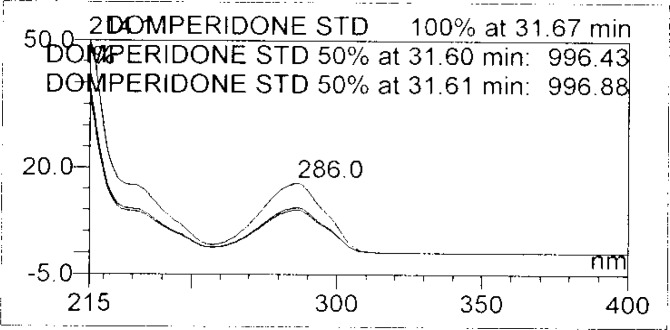
Purity Plot of Domperidone in 1M HCl

**Fig. 9:**
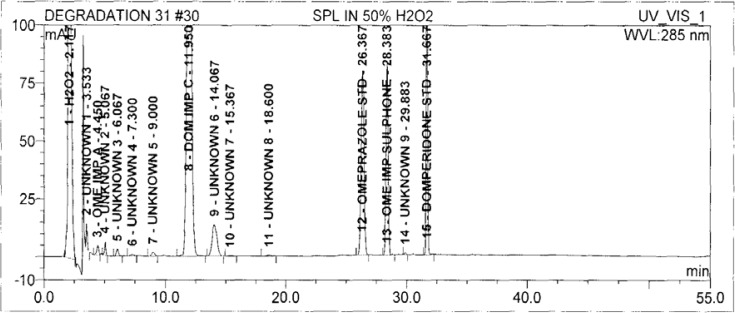
The typical Chromatogram of Sample in 50% H_2_O_2_

**Fig. 9a:**
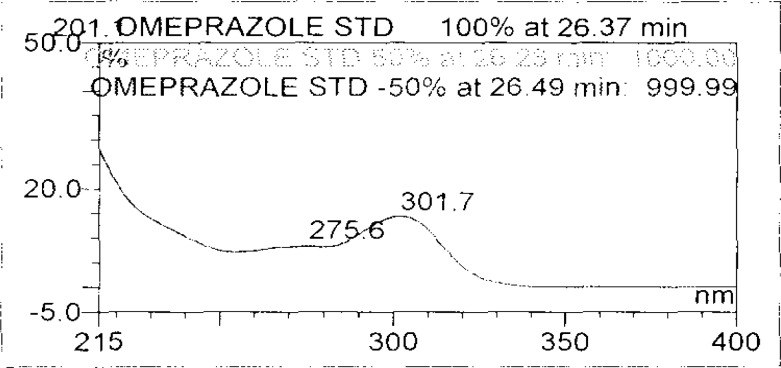
Purity Plot of Omeprazole in 50% H2O2

**Fig. 9b:**
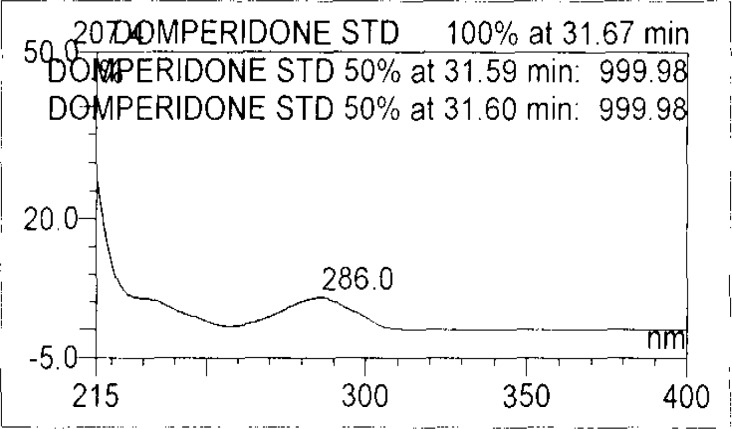
Purity Plot of Domperidone in 50% H_2_O_2_

**Fig. 10:**
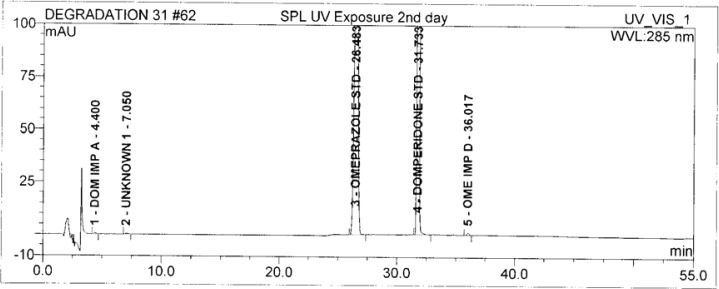
The typical Chromatogram of Sample in Heat

**Fig. 10a:**
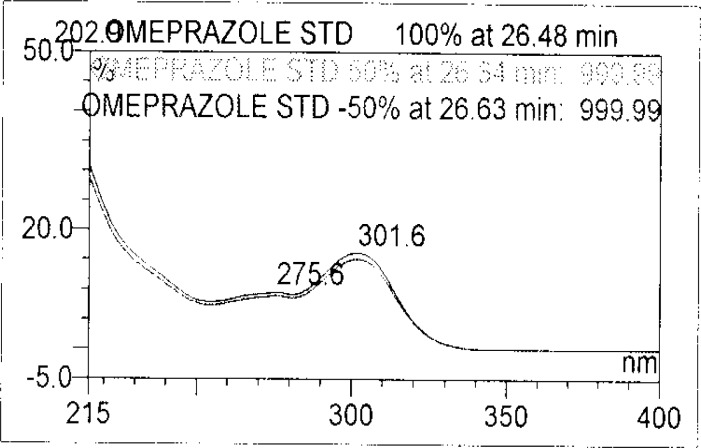
Purity Plot of Omeprazole in Heat

**Fig. 10b:**
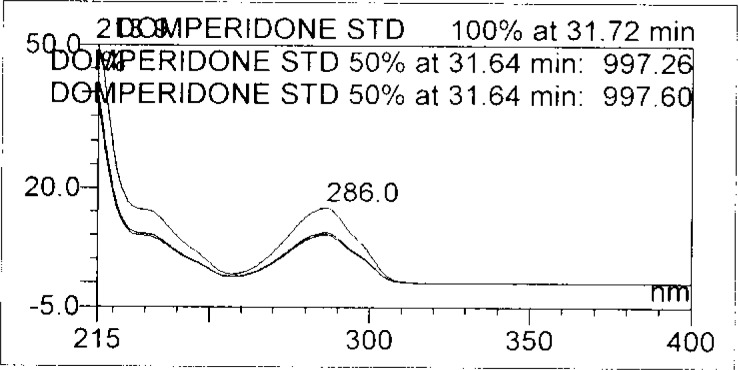
Purity Plot of Domperidone in Heat

**Fig. 11:**
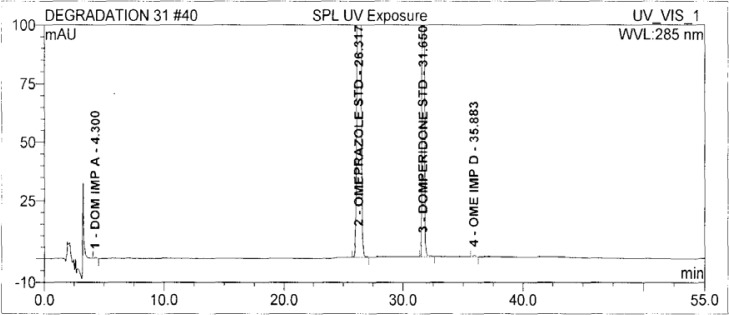
The typical Chromatogram of Sample in UV

**Fig. 11a:**
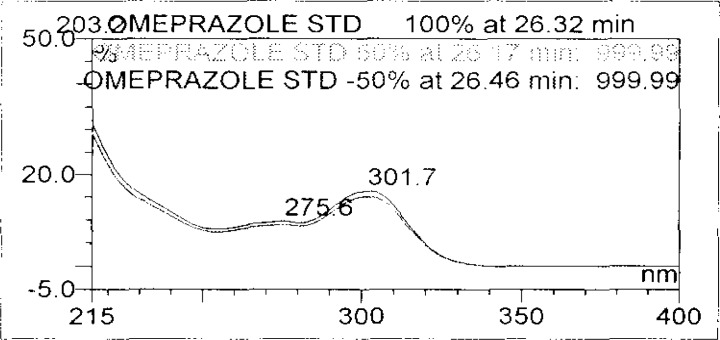
Purity Plot of Omeprazole in UV

**Fig. 11b:**
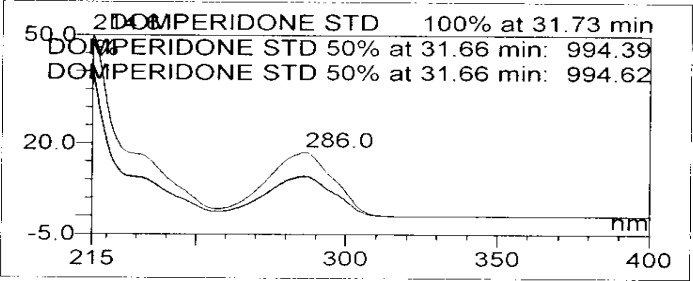
Purity Plot of Domperidone in UV

**Fig. 13:**
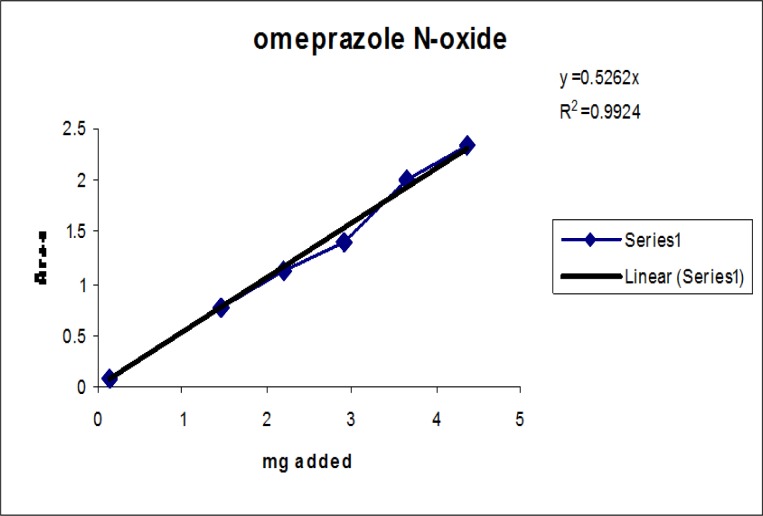
Linearity plot of Omeprazole N Oxide

**Fig. 14:**
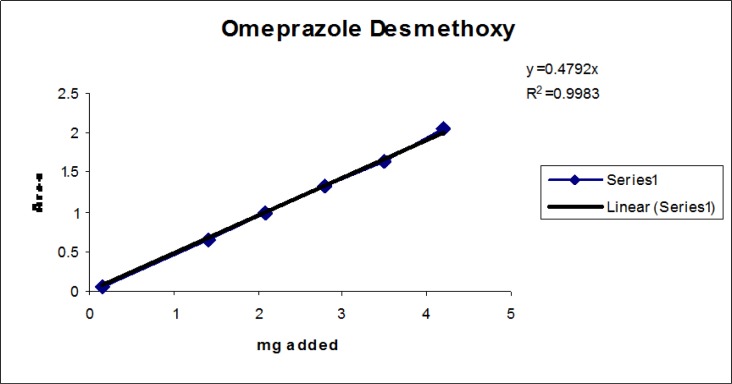
Linearity plot of Omeprazole Desmethoxy impurity

**Fig. 15:**
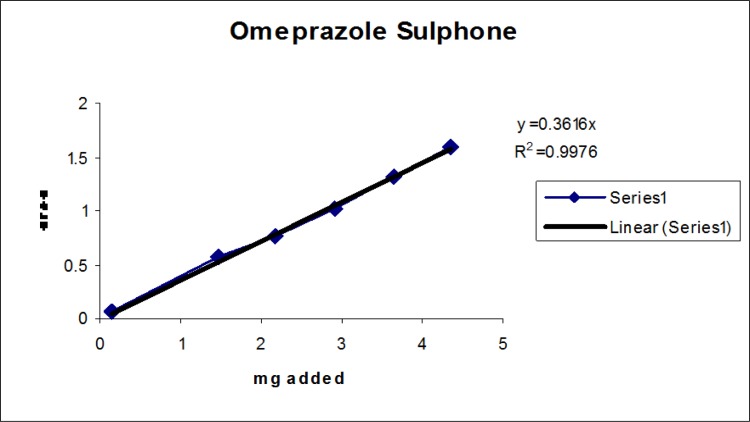
Linearity plot of Omeprazole Sulphone

**Fig. 16:**
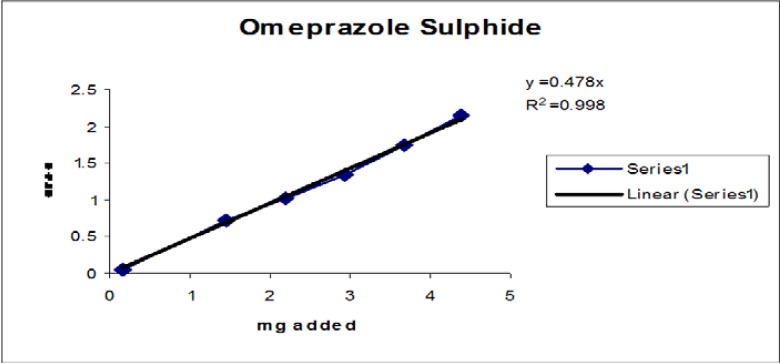
Linearity plot of Omeprazole Sulphide

**Fig. 17:**
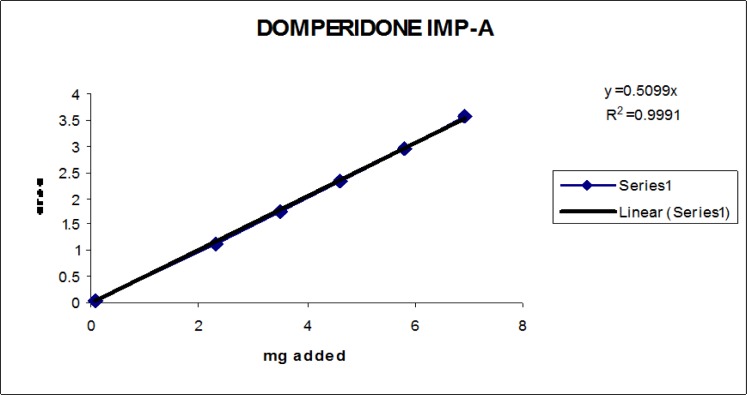
Linearity plot of Domperidone Imp-A

**Fig. 18:**
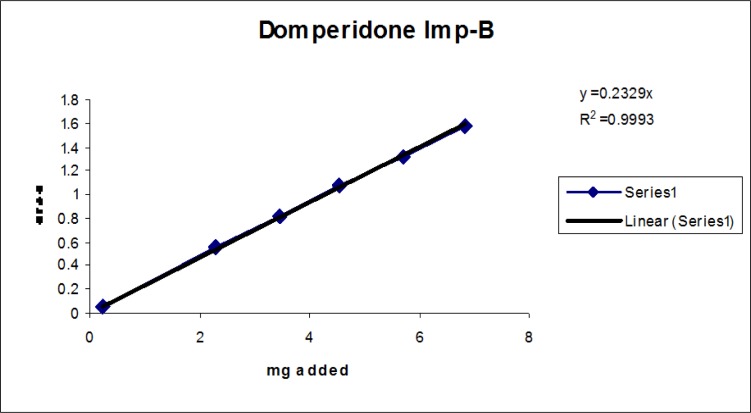
Linearity plot of Domperidone Imp-B

**Fig. 19:**
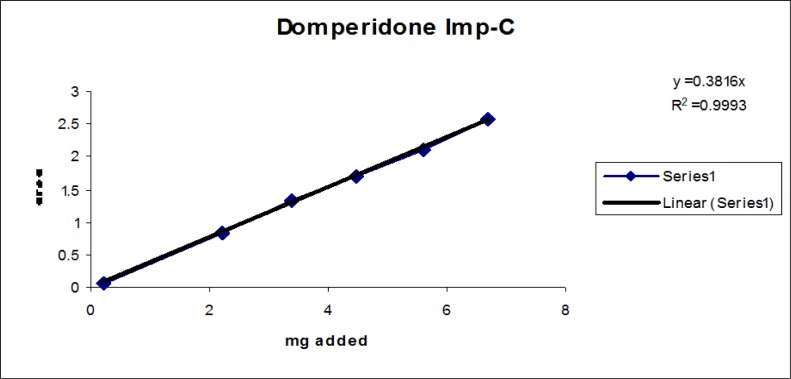
Linearity plot of Domperidone Imp-C

**Fig. 20:**
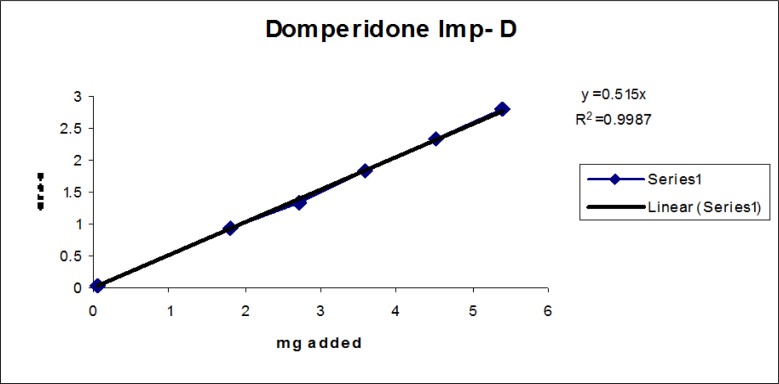
Linearity plot of Domperidone Imp-D

**Tab. 1.** Relative response factor results

**S.No.**	**Name**	**RRF**
1	Omeprazole N Oxide	1.67
2	Omeprazole C 789	1.61
3	Omeprazole Sulphone	1.11
4	Omeprazole des methoxy	1.33
5	Omeprazole Sulphide	1.49
6	Domperidone Impurity A	0.88
7	Domperidone Impurity B	0.74
8	Domperidone Impurity C	0.79
9	Domperidone Impurity D	0.99
10	Domperidone Impurity F	0.81

**Tab. 2.** Results of forced degradation study

**Stress Condition**	**Drug product**		

	**Peak purity match of Omeprazole**	**Peak purity match of Domperidone**	**% of degradation**
Acid Hydrolysis (1 N HCl)	999	995	47.3
Base Hydrolysis (1 N NaOH)	1000	993	5.3
Peroxide Oxidation (50% H2O2 reflux for 30 min)	1000	1000	52.0
Head Stress study at 105 °C for 48 hours	1000	996	0.91
UV stress study (254 nm) for 48 hours	1000	995	0.23

Remarks: Peak purity of Active peaks (OZ & DP) in stressed samples should be not less than 990 (factor no. as per HP Chemstation software).

**Tab. 3.** Results of Test method Precision

**Omeprazole Impurities**					

**TEST**	**Omeprazole Impurity C (%)**	**Omeprazole Impurity N Oxide (%)**	**Omeprazole Impurity demethoxy (%)**	**Omeprazole Impurity sulphone (%)**	**Omeprazole Impurity Sulphide (%)**
1	0.30	0.33	0.28	0.30	0.32
2	0.30	0.33	0.28	0.30	0.32
3	0.29	0.33	0.28	0.29	0.34
4	0.30	0.34	0.28	0.30	0.35
5	0.29	0.33	0.28	0.29	0.34
6	0.31	0.34	0.28	0.30	0.35
Mean	0.30	0.33	0.28	0.30	0.34
%RSD	1.77	0.88	0.91	1.21	3.85

**Domperidone impurities**					

1	0.33	0.39	0.31	0.41	0.28
2	0.33	0.39	0.32	0.42	0.28
3	0.33	0.38	0.31	0.41	0.29
4	0.34	0.39	0.32	0.42	0.29
5	0.33	0.38	0.32	0.41	0.28
6	0.34	0.39	0.32	0.42	0.29
Mean	0.34	0.39	0.32	0.42	0.28
%RSD	1.39	1.07	1.32	1.05	2.53

**Tab. 3.** Results of Limit of Detection and Quantification of impurities

**Component**	**Concentration with respect to sample concentration at**	

	**LOD**	**LOQ**
Domperidone Impurity A	0.0019	0.005
Domperidone Impurity B	0.0052	0.015
Domperidone Impurity C	0.0052	0.015
Domperidone Impurity D	0.0018	0.005
Domperidone Impurity F	0.0059	0.017
Omeprazole Impurity C	0.0055	0.016
Omeprazole impurity N-Oxide	0.0051	0.015
Omeprazole impurity Desmethoxy	0.0051	0.015
Omeprazole impurity Sulphone	0.0052	0.015
Omeprazole impurity Sulphide	0.0051	0.015

**Tab. 5.** Results of Impurities Recovery study on formulation sample

**Name of impurity**	**% mean recovery**
Domperidone Impurity A	108.54
Domperidone Impurity B	88.65
Domperidone Impurity C	100.15
Domperidone Impurity D	101.64
Domperidone Impurity F	98.66
Omeprazole Impurity C 789	97.45
Omeprazole impurity N-Oxide	95.9
Omeprazole impurity Desmethoxy	99.39
Omeprazole impurity Sulphone	101.99
Omeprazole impurity Sulphide	104.37

## References

[1] Aydan Y, Rustii O (2006). Specific H^+^/K^+^-ATPase inhibitors decreased contractile responses of isolated rat vas deferens. Pharmacol Res.

[2] Shimatani T, Inoue M, Kuroiwa T, Xu J, Mieno H, Tazuma S (2006). Acid suppressive effect of Generic Omeprazole, comparison of 3 brands of Generics with Omeprazole. Dig Liver Dis.

[3] Ahmad N, Keith-Ferris J, Gooden E, Abell T (2006). Making a case for Domperidone in the treatment of gastro intestinal motility disorders. Curr Opin Pharmacol.

[4] Drug Bank Mechanism of action of Domperidone.

[5] Naser LR, Keven CB, Angela DMK (2006). A simple sensitive bioanalytical assay for simultaneous determination of omeprazole and its 3 major metabolites in human blood plasma using RP-HPLC after a simple liquid-liquid extraction procedure. J Chromatogr B.

[6] Ribani M, Collins CH, Bottoli CB (2007). Validation of chromatographic methods: evaluation of determination detection and quantification limits in the determination of impurities in Omeprazole. J Chromatogr A.

[7] Danica A, Novovic D, Katarina KR, Valentina M (2004). Densitometric determinaion of Omeprazole, Pantoprazole and their impurities in Pharmaceuticals. J Planar Chromatogr.

[8] (2005). Guidance on validation of analytical procedure.

